# Does the central dogma still stand?

**DOI:** 10.1186/1745-6150-7-27

**Published:** 2012-08-23

**Authors:** Eugene V Koonin

**Affiliations:** 1 National Center for Biotechnology Information, National Library of Medicine, National Institutes of Health, Bethesda, MD, USA

## Abstract

**Abstract:**

Prions are agents of analog, protein conformation-based inheritance that can confer beneficial phenotypes to cells, especially under stress. Combined with genetic variation, prion-mediated inheritance can be channeled into prion-independent genomic inheritance. Latest screening shows that prions are common, at least in fungi. Thus, there is non-negligible flow of information from proteins to the genome in modern cells, in a direct violation of the Central Dogma of molecular biology. The prion-mediated heredity that violates the Central Dogma appears to be a specific, most radical manifestation of the widespread assimilation of protein (epigenetic) variation into genetic variation. The epigenetic variation precedes and facilitates genetic adaptation through a general ‘look-ahead effect’ of phenotypic mutations. This direction of the information flow is likely to be one of the important routes of environment-genome interaction and could substantially contribute to the evolution of complex adaptive traits.

**Reviewers:**

This article was reviewed by Jerzy Jurka, Pierre Pontarotti and Juergen Brosius. For the complete reviews, see the Reviewers’ Reports section.

## The Central Dogma and apparent nonexistence of reverse translation

**Figure 1:**
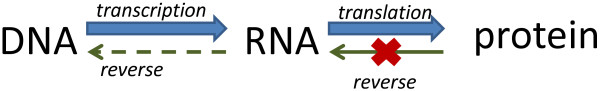
The Central Dogma of Molecular Biology.

There are very few firm principles in biology. It is often said, in one form or another, that the only actual rule is that there are no rules, i.e. exceptions can be found to every ‘fundamental’ principle if one looks hard enough. The principle known as the Central Dogma of molecular biology seems to be an exception to this ‘ubiquitous exception’ rule [1]. The Central Dogma was conjured by Francis Crick in response to the discovery of reverse transcription [2,3], when it became clear that the RNA to DNA information transfer was an integral part of the life cycle of retro-transcribing genetic elements (subsequent developments demonstrated the broad occurrence of reverse transcription in cells [4,5]) (Figure 1). Crick realized that, all its biologically fundamental implications notwithstanding, reverse transcription was essentially business as usual, i.e. interconversion of different forms of nucleic acids on the basis of universal rules of base complementarity. The central dogma places the actual ‘exclusion principle’ at another stage of biological information transfer, translation. Thus, ‘There is no information transfer from protein to nucleic acid’, postulates the Central Dogma. This postulate is not based on any physical law (in principle, all reactions involved in translation are reversible) but rather on the design of the translation system that hampers reverse translation. Information flow back from a protein sequence to the cognate nucleic acid sequence by reverse translation would require an elaborate sequence of reactions that are not known to exist in any life forms. The two fundamental steps would be: i) recognition of nucleotide triplets (tRNA anticodons) by amino acid residues within a polypeptide chain, ii) joining of these triplets into an RNA molecule.

There is no reason to consider the Central Dogma a physical ‘exclusion principle’. However, it appears to be a fundamental ‘biological law’ that is deeply rooted in the molecular setup of the information flow in all cells. Indeed, an entire, distinct system of ‘reverse information transfer’ would have been required to channel information back from the protein to nucleic acid sequence. Furthermore, given the degeneracy of the genetic code, reverse translation could only be a stochastic process and would entail major loss of information (but also potential generation of new information). There is no trace of a reverse translation system in any of the thoroughly characterized model organisms.

## Protein-based analog inheritance: the prions

Reverse translation has not been discovered so far and seems extremely unlikely to ever be discovered. However, the Central Dogma is not about a specific molecular mechanism but rather about information flow: not about the (im)possibility of reverse translation but rather about the (non)existence of information flow from protein to nucleic acid. Is it conceivable that this channel of information transfer is after all not fully closed but the underlying molecular mechanisms are completely different from the hypothetical reverse translation?

Enter prions. The entities that eventually became known as prions were first discovered as agents of slow, devastating neuro-degenerative diseases (spongiform encephalopathies), the relatively common scrapie in sheep and the rare Kuru and Creutzfeld-Jacob diseases in humans [6,7]. The agents of these diseases showed extremely unusual properties, in particular extraordinary resistance to treatment that inactivates even the smallest nucleic acid molecules such as high-dose UV irradiation [8]. The history of research on the agents of spongiform encephalopathies involved numerous false leads in the persistent quest for a conventional virus or an unusual nucleic acid-containing agent linked to these diseases [9,10]. Eventually, a series of meticulous experiments by Prusiner and colleagues (winning the 1997 Nobel Prize in Physiology or Medicine) has demonstrated beyond doubt that an iconoclastic hypothesis originally proposed by Griffith [11] held true: the infectivity of the scrapie agent was completely protein-mediated [12-15].

**Figure 2:**
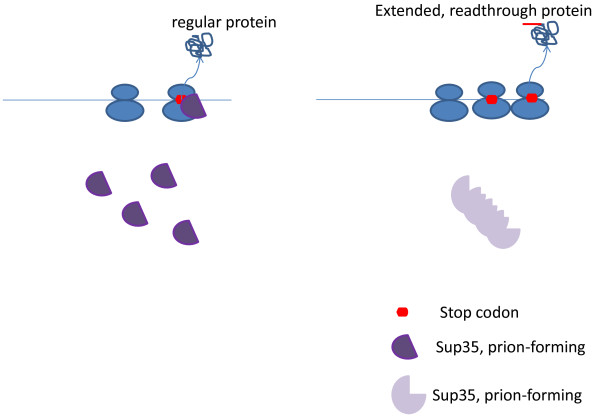
Prion (Sup35)-mediated generation of epigenetic variation.

The protein-only infectious agents and subsequently discovered factors of epigenetic heredity received the name prions [15,16]. The prion proteins assume two distinct conformations one of which is soluble whereas the other one aggregates to form amyloid-like fibrils [17-19]. The amyloid-forming conformer possesses self-propagating properties: once a prion molecule assumes this conformation, it interacts with other molecules in the soluble conformation and induces their conversion to the amyloid-forming conformation, much like Ice-9 in Kurt Vonnegut’s *Cat’s Cradle*[20] (Figure 2). Thus, prions are agents of analog heredity, in a sharp contrast to the mainstream, nucleic acid-mediated digital heredity.

A seminal discovery that greatly facilitated further study of prions was the demonstration that prions exist not only in animals but also in yeast where they mediate epigenetic inheritance of phenotypic traits [21-26]. To date, about two dozen yeast prions have been characterized to a varying degree of molecular detail but screening for prion inheritance indicates that many more exist [25-29]. The distinctive structural feature of prion proteins is the presence of a disordered prion-determining domain that triggers the conformational transition [30-32]. The prion conformation forms spontaneously at a low frequency (on the order of 10^-6^) [33,34]. Switching to and from the prion state increases in rate under stress [35-37], and mutants have been isolated, in particular in heterologous prion genes, with much higher frequency of prion formation [38,39].

The best characterized yeast prion is [PSI+], the Sup35 translation-termination factor [40]. In the prion strains that have been shown to be common in nature and conserved in diverse fungi [29], most of the Sup35 is sequestered in amyloid, the result being a dramatic increase in the rate of termination codon readthrough [41]. The aberrant readthrough proteins induce a variety of phenotypes of which a significant fraction are beneficial under selective conditions [42,43]. Thus, the Sup35 prion is a catalyst of protein variation that is often discussed in terms of the bet-hedging adaptation strategy [29,37,44]. Every grown colony of yeast will contain several cells with the prion. If, under stress, the variation engendered by the prion turns out to be deleterious, only a few cells will perish without perceptible fitness consequence to the entire colony. However, if a beneficial variant emerges, the prion-carrying cells have the potential to take over the colony ensuring survival under adverse conditions. It is less clear whether prions other that [PSI+] (Sup35) promote phenotypic variability but the recent results with the [MOT3+] prion, a repressor of transcription, revealed properties generally mimicking those of Sup35 [29]. Also, many of the described prions are proteins involved in transcription and RNA processing which is compatible with their role in generating variation [28,29]. Furthermore, the findings that prion formation is induced by stress [35-37] and that prions accurately segregate between daughter cells during cell division through the action of the molecular chaperone HSP104 [45-48] strongly suggest that prions are at least partially adaptive [29] rather than being simply a ‘molecular disease’ [49,50].

The most striking observation on prion-mediated epigenetic inheritance is that it can be turned into prion-independent genetic inheritance with a relative ease, a phenomenon denoted genetic assimilation of an epigenetically inherited trait. Assimilation can be achieved simply by meiotic reassortment of pre-existing genetic variation [29,42,43]. The relatively low frequency of assimilation implies that several mutations are required. The assimilation phenomenon has not been investigated in much detail, and in particular, no genome sequences of the assimilating strains have been reported, so it remains unknown what are the exact mutations that lead to fixation of the respective traits in a prion-independent form. The most straightforward possibility is that during assimilation genetic variation recapitulates the variation that is unmasked by the prion, e.g. the readthrough variants induced by the Sup35 prions. However, it cannot be ruled out that the same phenotypic effects ensue, at least in part, from different mutations. Regardless of the exact mechanisms, prions clearly violate the Central Dogma by enabling the information flow from proteins to the genome.

## The general look-ahead effect

**Figure 3:**
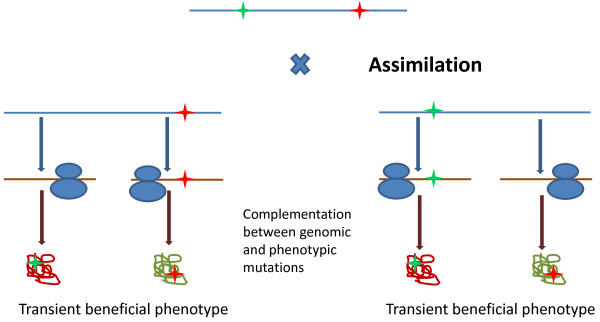
The general look-ahead effect: information flow from protein sequence to genome via assimilation of epigenetic variation.

In general terms, what prions do, is extremely simple: they buy time for the cell to accumulate the beneficial combination of mutations through recombination and possibly new mutations as well. This appears to be a specific manifestation of a most general phenomenon (Figure 3). Any phenotypic variation in protein sequence or structure, such as spontaneous error of transcription, splicing, RNA editing or translation, capacitation of hidden genetic or phenotypic variability or protein misfolding, that is beneficial either on its own or combined with another mutation (i.e. depending on the genetic background) can potentially become hereditary through the ‘look-ahead effect’ [51], i.e. by allowing the organism time to generate the required genotype by recombination or to generate the required mutation(s) *de novo*. In other words, a phenotypic and a genomic mutation complement each other to produce a transient beneficial phenotype (Figure 3). The rate of amino acid misincorporation is orders of magnitude greater than the replication error rate, so any cell contains numerous variant proteins [52,53]. These phenotypic mutations might underlie evolution of complex traits that require more than one mutation (a simplest example of a potentially beneficial structural feature of proteins that requires two mutations is a new disulfide bond). Direct experimental study of the look-ahead effect is still lacking but population-genetic models indicate that phenotypic mutations can be sufficient for alleles carrying only one mutation required for a trait dependent on two mutations to spread in a population provided the selective advantage of the combination of two mutations is high enough [51]. Furthermore, the error rate of translation increases under stress [54,55] with the implication that the look-ahead effect could be particularly important to generate stress-resistant phenotypes – and eventually genotypes. Moreover, agents that specifically induce mistranslation, such as streptomycin, also cause a mutator phenotype via mistranslation-induced mutagenesis [56], directly demonstrating the coupling of epigenetic and genetic variation [57]. The look-ahead effect can be similarly potentiated by errors of transcription, splicing or RNA editing. Although in these cases, the actual change occurs in the RNA sequence, the net result is the same: information potentially can be transferred from a protein sequence back to the genome.

A related phenomenon is capacitation of hidden variation. The best characterized capacitor is the molecular chaperone HSP90 that prevents misfolding of variant proteins arising either from genomic or from phenotypic mutations [58,59]. This hidden variation is unmasked and results in multiple phenotypic effects when the function of HSP90 is compromised, in particular under environmental stress [60-62]. Even beyond capacitating hidden variation, inactivation of HSP90 causes large-effect mutations, such as aneuploidy, that on some occasions become adaptive under stress [63]. Screening for hidden variation capacitors in yeast has shown that hundreds of proteins possess capacitor properties [64]. It seems likely that release of hidden variation, both genetic and phenotypic, is a regulated form of stress response. In effect, capacitation is an amplifier of the look-ahead affect. This phenomenon links robustness and evolvability of evolving biological systems by ensuring robustness under normal conditions but promoting evolvability under stress.

Thus, the look-ahead effect of phenotypic mutations, whether in its basic form or enhanced by capacitors, prions and possibly other factors, seems to be an important, possibly central factor of evolvability, particularly with respect to the evolution of complex adaptive traits. This route of evolution plainly violates the Central Dogma.

## Violation of the Central Dogma and Lamarckian inheritance

The Central Dogma is often linked with the purported (im)possibility of Lamarckian inheritance [65]. Indeed, if the information flow from proteins to the genome existed, it could be imagined that environmental cues could have a directed effect on genomes. However, a deeper examination of the available data shows that violation of the Central Dogma is not necessary for Lamarckian evolution and conversely that not all evolutionary phenomena that involve such violation are necessarily Lamarckian. The reality of full-fledged Lamarckian evolution that is completely based on standard complementary interactions between nucleic acid strands has been convincingly demonstrated by the discovery and subsequent study of the CRISPR-Cas adaptive immunity system in archaea and bacteria [66-69]. This system specifically responds to an environmental cue (virus or plasmid invasion) by directed changes in the genome that provide adaptation to this particular cue (immunity). The integration of Piwi RNAs into animal germ line providing defense against specific transposable elements seems to fit the definition of Lamarckian evolution equally well [70,71]. A much broader range of phenomena including the pervasive horizontal gene transfer in prokaryotes and stress-induced mutagenesis that exists in most if not all cellular life forms combine clear Lamarckian elements with substantial stochasticity and could be most appropriately classified as quasi-Lamarckian [67,72]. The general look-ahead effect that contributes to evolution via information flow from proteins to the genome seems to belong in the quasi-Lamarckian category. There is a Lamarckian component to this form of evolution because it is stimulated by environmental stress. However, there are also major stochastic and selection contributions given that variation is undirected and unrelated (as far as currently known) to specific environmental cues, so that the fittest variants survive by selection.

## Conclusions

The Central Dogma of molecular biology is refuted by genetic assimilation of prion-dependent phenotypic heredity. This phenomenon is likely to be the tip of the proverbial iceberg, a specific, most dramatic manifestation of a major facet of evolution that I denoted here ‘general look-ahead effect.’ Even more generally, the entire spectrum of epigenetic variation, in particular various modifications of DNA, chromatin proteins and RNA, potentially can be similarly assimilated by evolving genomes. It is interesting to note that genetic assimilation of phenotypic adaptation had been predicted [73] and then experimentally demonstrated by Waddington in classic experiments on *Drosophila* over half a century ago [74]. Obviously, no molecular details of this process could be deciphered at the time.

The specific cases of prions and capacitation of cryptic variation discussed here have been discovered and explored in eukaryotic model systems. Although specific mechanisms of information transfer from proteins to genome, such as the prions, indeed might be more common or even unique in eukaryotes, the look-ahead effect as such seems to be a general feature of all evolving life forms and might be particularly important in viruses for which the standing variation in populations is particularly high.

Thus, the Central Dogma of molecular biology is invalid as an ‘absolute’ principle: transfer of information from proteins (and specifically from protein sequences) to the genome does exist. This is not to deny that the Central Dogma does capture the principal route of information transfer in biology: the main flow information does follow the path in Figure 1, and elaborate mechanisms ensuring acceptable fidelity operate on each step. And, there is a major discontinuity between the levels of RNA and protein because during translation because the coupling of amino acids with the cognate tRNAs does not involve direct recognition but rather requires dedicated enzymes, aminoacyl-tRNA synthetases, that recognize and connect the partners. The opposite direction of information flow, from proteins to the genome, is asymmetrical (not a simple reversion), much more modest quantitatively and intrinsically stochastic but nevertheless appears to be important in evolution.

## Competing interests

The author declares that he has no competing interests.

## Author’s contributions

EVK wrote the manuscript.

## Reviewers’ reports

Reviewer 1: Jerzy Jurka, Genetics Information Institute

This is a straightforward paper exploring important implications of prion-mediated heredity for the Central Dogma.

I have only some minor comments:

(1) It would be useful to quote the article Alain E. Bussard on the same subject [75], and to highlight the major new arguments introduced in the current article.

**Author’s response:***Bussard’s article appears to be something of a misnomer in that the author puts the Central Dogma in the title but does not really examine it with respect to the properites of prions. His article instead explores the Lamarckian features of the prion inheritance and certainly is of interest for its historical aspects.*

(2) I believe that the author should also comment on RNA editing in the “look-ahead” section; e.g. [76].

**Author’s response:***This comment is appreciated, an interesting point is brought up here. Although as far as RNA editing or transcriptional errors are involved, the actual mutating entity is RNA rather than protein, the net effect on heredity is the same as with translation errors. Effectively, a version of the look-ahead effect indeed could be engendered by RNA editing, with stochastic editing creating variation that can be subsequently fixed in the genome through convergent genetic variation. I included a statement to that effect in the revised version of the manuscript. The same pertains to errors of transcription. Unfortunately, the report of extensive editing by Li et al.*[76]*has been compromised to such an extent*[77-79]*that it has become difficult to interpret these observations.*

(3) Finally, I recommend including the article by Sergey G. Inge-Vechtomov *et al.*[16], that includes a unique historical perspective on “non-inherent variability” dating back to Kirpichnikov.

**Author’s response:***I cited the article by Inge-Vechtomov et al. as a review but my reading again is that this is about protein-based heredity not violations of the Central Dogma*.

Reviewer 2: Pierre Pontarotti, Universités de Provence et de la Méditerranée

In this review/outlook, the author studied the assumption that the information could be originated from protein to protein and from phenotypes to DNA. The author also highlighted that “violation of the central dogma” and the “epigenetics trans generational inheritance” are two different phenomena, even if sometime, they could be connected. Although this point seems to be obvious, this clarification is essential (I meet several scientists mixing the two concepts). In my opinion, I think that the main question asked by the author: “Does the central dogma still stand?” is opportune. Investigators really need to transgress scientific dogmas. But we will still need to propose robust approaches to test new hypotheses.

Reviewer 3: Juergen Brosius, University of Muenster

The author questions the Central Dogma of Molecular Biology, because structural modification of prion-like proteins might have a (more global) effect on the expression and even structure of gene products. The idea is based on data obtained with studies of yeast prion-like proteins, for example, the Sup35 protein that normally acts as a translation termination factor. However, when sequestered in amyloid, hence insoluble and inactive, its deficiency allows for readthrough of termination codons in messenger RNAs.

The author places such strategies of increasing evolvability into the category of quasi-Lamarckian evolution. Like most phenomena in biology, similarity to the Lamarckian mode of evolution lies somewhere on a continuum ranging from barely apparent to very strong. In my opinion, the possible elongation of polypeptides is at the very weak end of the continuum of Lamarckian mode of evolution, not too remote from random mutations of nucleotides, since this process is almost equally non-directed.

**Author’s response:***I am not going to strongly argue this point. Thea ‘quasi-Lamarckian’ mechanisms certainly belong to the continuum of evolutionary phenomena, from stochastic to deterministic ones*[67]*. The Lamarckian character of this readthrough is not the focus of the present article which is about information transfer from protein to genome; the readthrough seems to capacitate such transfer.*

In addition, most S. cerevisiae 3′-UTRs tend to be short, typically in the size range of 50 to 200 nucleotides, with a median length of 121 nt [80]. Should a C-terminal ORF extension truly be part of a ‘look ahead effect’ [50] for times of stress, might one not observe distal to the bona fide stop codons a slightly higher conservation of the first two positions in the respective codons?

**Author’s response:***This is a really, really interesting idea. Yes, in principle, one should expect some degree of purifying selection in the sequences downstream of stop codons. However, because readthrough is not frequent, the effect could be quite weak so that its detection would require sophisticated statistical analysis of large data sets. To the best of my knowledge, no one has shown that such conservation does not exist (a difficult task as well). This seems to be well worth investigating.*

Furthermore, messenger RNAs transcribed from genes containing mutations that generate aberrant extended 3′ untranslated regions are degraded by nonsense mediated decay (NMD) [81-83]. Even if NMD was suppressed by an additional stress induced mechanism, the C-termini of proteins usually are the least conserved parts of a protein and often can be altered or extended without functional consequences [84]. Once more, the action of prions apparently do not have an effect on their own expression or C-terminal extension. Due to this undirected nature, I would place prion formation to the weak end of the continuum concerning quasi-Lamarckian mode of evolution. Something similar could be said about modification of nucleic acids, most prominently methylation of DNA in control regions of genes, as this process seems to be not specifically directed. However should the link between prenatal nutrient deprivation in humans and adiposity in later life - in conjunction with the findings that reduced methylation of the insulin like growth factor II gene (IGF2) is the underlying molecular mechanism for this effect – become substantiated, at least some of these epigenetic effects due to methylation changes could be placed closer to the opposite end of the continuum [85-87]. Animal studies will be essential to rigorously test these observations initially made in human populations. The case of the prokaryotic CRISPR-cas system of defense against mobile elements including plasmids and viruses is an interesting and much stronger case as covered by the author in a previous publication [67]. Nevertheless, a stochastic event and not the need for viral defense lead to integration and antisense transcription of part of the invader’s genome. The fortuitous beneficiary effect of, e.g., antiviral protection was of selective advantage and became fixed. As mentioned in the review of this Koonin/Wolf article, in my view, the examples that most closely resemble Lamarckism or quasi-Lamarckism stem from several ongoing transitions in our own lineage, namely vertical and horizontal transmission of memes [88,89] and at the level of genes through our potential (not yet realized) to direct acquired knowledge about genotype/phenotype relationships into our own genome in a precise and specific manner via genetic engineering [90-92].

**Author’s response:***These comments are appreciated. I find memetics to be of much interest and promise. However, in my view, this field of enquiry is outside evolutionary biology sensu strictu.*

The Central Dogma of Molecular Biology is, in my opinion, still untouched as there is no reverse translation. This would change if a mutation in a given protein including a translational readthrough beyond the stop codon directly would lead to a nucleotide change that converts said stop codon into one that encodes an amino acid. My own problem with the Central Dogma of Molecular Biology is different, more trivial, and based on the depiction of DNA and not RNA topping the hierarchy [93].

**Author’s response:***This is a key conceptual point that is addressed in the main text of the present article but is worth pondering again. True, to the best of our knowledge, there is no reverse translation but this is not what the Central Dogma is about. Quoting Crick*[1]*: ‘The central dogma of molecular biology deals with the detailed residue-by-residue transfer of sequential information. It states that such information cannot be transferred back from protein to either protein or nucleic acid.’ So Crick was fully explicit in formulating the Central Dogma as a ‘law’ of information transfer in biological systems not as a statement about specific reaction paths. It is remarkable that, although evolution failed to find ways to reverse transcription, it has found means to circumvent this irreversibility through completely different mechanism, and so after all, to reverse the direction of the information flow. As for the “more trivial” aspect, it is certainly indisputable that the diversity of RNA roles in biology, in particular in shaping genomes* via *retrotransposition, was vastly under-appreciated 40 years ago (and might not be fully appreciated yet).*
